# Fractionation of Hepatic Nonparenchymal Cells

**DOI:** 10.1100/tsw.2002.283

**Published:** 2002-05-16

**Authors:** John Graham

**Affiliations:** School of Biomolecular Sciences, Liverpool John Moores University, UK

**Keywords:** liver cells, hepatocytes, parenchymal cells, nonparenchymal cells, stellate cells, Kupffer cells, OptiPrep^TM^, iodixanol, density barrier

## Abstract

The majority of parenchymal cells from mammalian liver cells can be removed by very low speed centrifugation (50 g) but a simple low-density barrier (1.096 g/ml) is required to remove the remaining parenchymal cells from the 50-g supernatant which contains all of the lower density nonparenchymal cells. Continuous gradients of Nycodenz can provide satisfactory resolution of Kupffer, stellate, and endothelial cells on an analytical basis but the separation of different cell types is not sufficient preparatively. Flotation through a low-density iodixanol barrier can, however, provide a satisfactory enrichment of the least dense nonparenchymal cell – the stellate cells.

**METHOD TYPE:** extraction, isolation, purification and separation

**SUB METHOD TYPE:** centrifugation

## INTRODUCTION

The fractionation of hepatic nonparenchymal (sinusoidal) cells (Kupffer cells, stellate cells, endothelial cells, etc.) on continuous metrizamide[1] and Nycodenz® gradients[2] has shown that the overlap in banding densities of these cells makes these gradients generally unsatisfactory as a stand-alone procedure to isolate the various cell populations.

Discontinuous gradients of iodinated media are widely used to provide a means of removing residual erythrocytes and parenchymal cell debris from nonparenchymal cell preparations and to prepare a stellate cell–rich fraction.

## Preparation of Nonparenchymal Cells

Parenchymal cells are routinely prepared by collagenase digestion of the liver using a tissue perfusion system. These cells are then separated from the nonparenchymal cells by differential pelleting at 50 *g* for 1–4 min. Although the nonparenchymal cells can be isolated from the 50-*g* supernatant, the yields are usually low. The most widely used procedure is to perfuse the liver with a mixture of collagenase and Pronase or endotoxin to destroy the parenchymal cells selectively (see Refs. 1 and 2 for details).

## Stellate Cells

These are the least dense of the nonparenchymal cells and can be floated effectively away from the rest of the cells. The low-density fraction may contain almost 80% stellate cells[2]. The actual density of the low-density layer used by different workers varies somewhat and some of these variations are presented in the Notes section

The following methods are adapted from Bøyum et al.[1] and from Brouwer et a.l[2]. The advantage of using OptiPrep™ rather than Nycodenz® is that the density solutions can be made up by dilution of OptiPrep™ directly with Gey's Balanced Salt Solution (GBSS), while Nycodenz® must be diluted with GBSS minus the NaCl to keep the osmolality below 300 mOsm.

## MATERIALS AND EQUIPMENT

- Gey's Balanced Salt Solution (GBSS): 7.0 g NaCl, 0.37 g KCl, 70 mg MgSO4.7H2O, 150 mg Na2HPO4.2H2O, 220 mg CaCl2.2H2O, 2.27 g NaHCO3, 30 mg KH2PO4, 210 mg MgCl2.6H2O, 1.0 g glucose dissolved in 1 l of water, gassed with 5% CO2/air; the pH should be 7.4
- OptiPrep™ (60% w/v, iodixanol)
- 40% (w/v) Iodixanol Working Solution (WS): mix 4 vol of OptiPrep™ and 2 vol of GBSS
- Plastic conical centrifuge tubes (50 ml)
- Plastic Pasteur pipette for overlayering
- Low-speed (temperature-controlled) centrifuge with swinging-bucket rotor

## METHODS

## Preparation of Nonparenchymal Cells

1. Suspend the crude nonparenchymal cells in approx. 10 ml of GBSS ($1–4 \times 10^8$ cells).
2. Add WS to the cell suspension so that the final concentration of iodixanol is 17% (w/v) iodixanol solution ($\rho$ = 1.096 g/ml).
3. Mix thoroughly but gently.
4. Layer approx. 2 ml of GBSS on top and centrifuge at 400 *g* for 15 min. at 20°C.
5. Allow the rotor to decelerate without the brake.
6. Collect the cells, which band at the interface between the GBSS and the 17% iodixanol.

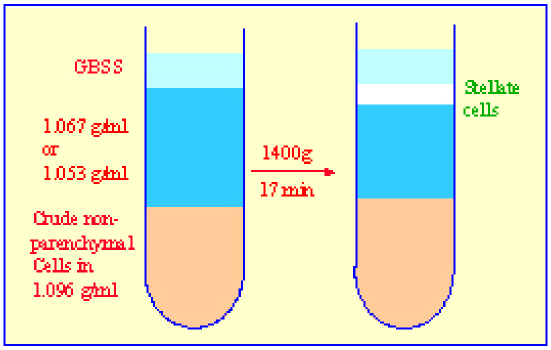

**FIGURE 1**. Isolation of hepatic stellate cells by flotation. GBSS = Gey's Balanced Salt Solution.

## STELLATE CELLS

1. Add WS to the cell suspension as described is steps 1–3 above.
2. Dilute W with GBSS to produce a solution containing 11.5% (w/v) iodixanol (see Note 1).
3. Layer 5 ml of this solution over the same volume of cell suspension (in 17% iodixanol); then layer 2 ml of GBSS on top (see Note 2).
4. Centrifuge at 1400 *g* for 17 min. at 20°C; allow the rotor to decelerate without the brake.
5. Collect the cells, which band at the interface between the GBSS and the 11.5% iodixanol (see Fig. 1).

## NOTES

1. This concentration of iodinated density gradient medium is equivalent to approx 1.067 g/ml and has been used both with Nycodenz®[1,2] and iodixanol[3,4]. A lower-density concentration (1.053 g/ml) was used by Cassiman et al.[5]. This is equivalent to approx. 9% (w/v) iodixanol.
2. In the method as described by Brouwer et al.[2], the cell suspension was placed in the top layer ($\rho$ = 1.067 g/ml) rather than the bottom layer ($\rho$ = 1.096 g/ml) and this is an alternative strategy.

## ACKNOWLEDGEMENTS

The author and TheScientificWorld wish to thank Axis-Shield PoC, AS, Oslo, Norway for their kind permission to adapt OptiPrep™ Application Sheet C24 in the preparation of this Protocol Article.
